# Imaging Features of Hepatocellular Carcinoma With Bile Duct Tumor Thrombus: A Multicenter Study

**DOI:** 10.3389/fonc.2021.723455

**Published:** 2021-11-05

**Authors:** Jun-Yi Wu, Li-Ming Huang, Yan-Nan Bai, Jia-Yi Wu, Yong-Gang Wei, Zhi-Bo Zhang, Mao-Lin Yan

**Affiliations:** ^1^ Department of Hepatobiliary Surgery, the Shengli Clinical Medical College of Fujian Medical University, Fuzhou, China; ^2^ Department of Hepatobiliary Pancreatic Surgery, Fujian Provincial Hospital, Fuzhou, China; ^3^ Department of Hepatobiliary Surgery, West China Hospital of Sichuan University, Chengdu, China; ^4^ Department of Hepatobiliary Surgery, The First Affiliated Hospital of Fujian Medical University, Fuzhou, China

**Keywords:** hepatocellular carcinoma, bile duct tumor thrombus, computed tomography, magnetic resonance imaging, intrahepatic bile duct dilation

## Abstract

**Objectives:**

There are still challenging problems in diagnosis of hepatocellular carcinoma (HCC) with bile duct tumor thrombus (BDTT) before operation. This study aimed to analyze the imaging features of HCC with B1–B3 BDTT.

**Materials and Methods:**

The clinicopathological data and imaging findings of 30 HCC patients with B1–B3 BDTT from three high-volume institutions were retrospectively reviewed. A total of 631 patients without BDTT who were randomly collected from each of the enrolled centers were recorded as the control group to analyze the differences in clinicopathological characteristics and imaging features between the two groups. A total of 453 HCC patients who underwent surgical treatment in the three institutions from January 2020 to December 2020 were collected for a blinded reading test as the validation group.

**Results:**

HCC patients with B1–B3 BDTT had more advanced tumor stages and adverse clinicopathological features. HCC lesions were detected in all patients, and intrahepatic bile duct dilation was observed in 28 (93.3%) patients with B1–B3 BDTT and 9 (1.43%) patients in HCC without BDTT. The intrahepatic bile duct dilation showed no enhancement at hepatic arterial phase (HAP) and no progressively delayed enhancement at portal venous phase (PVP), but it was more obvious at PVP on CT. In the reports of the 30 HCC patients with B1–B3 BDTT generated for the image when the scan was done, BDTT was observed in all 13 B3 patients and 3 of 12 B2 patients, but none of the 5 B1 patients. Fourteen patients were misdiagnosed before surgery. However, when using intrahepatic bile duct dilation in HCC patients as a potential biomarker for BDTT diagnosis, the sensitivity and specificity for BDTT diagnosis were 93.33% and 98.57%, respectively. The blinded reading test showed that intrahepatic bile duct dilation in CT and MRI scans could be for separating HCC patients with B1–B3 BDTT from HCC patients without BDTT.

**Conclusions:**

The HCC lesions and intrahepatic bile duct dilation on CT or MRI scans are imaging features of HCC with BDTT, which might facilitate the early diagnosis of B1–B3 BDTT.

## Introduction

Hepatocellular carcinoma (HCC) is the sixth most common cancer and the third leading cause of cancer-related deaths worldwide (1). HCC often invades the portal vein and forms a tumor thrombus. HCC with bile duct tumor thrombus (BDTT) is uncommon, with an incidence between 0.53% and 12.9% (2–5).

Previous studies have attempted to explore the clinico-pathological characteristics and surgical treatment of HCC with BDTT (6–10). Hepatectomy is generally considered the preferred treatment for HCC with BDTT. Therefore, accurate diagnosis and surgical treatment are important to improve survival. Both computed tomography (CT) and magnetic resonance imaging (MRI) have diagnostic value for HCC with BDTT. There remain challenges in the diagnosis of HCC with BDTT before operation. According to the classification proposed by the liver cancer study group of Japan, BDTT was classified as B1–B4 (11). Several reports focusing on the CT or MRI features of HCC with B4 BDTT have been described (12–17).

However, to the best of our knowledge, the imaging features of HCC with B1–B3 BDTT have not been reported in the literature. Thus, the purpose of our study was to analyze the CT or MRI characteristics of HCC with B1–B3 BDTT to have a better understanding and early diagnosis of this disease.

## Materials and Methods

### Patient Population

**Figure 1:**
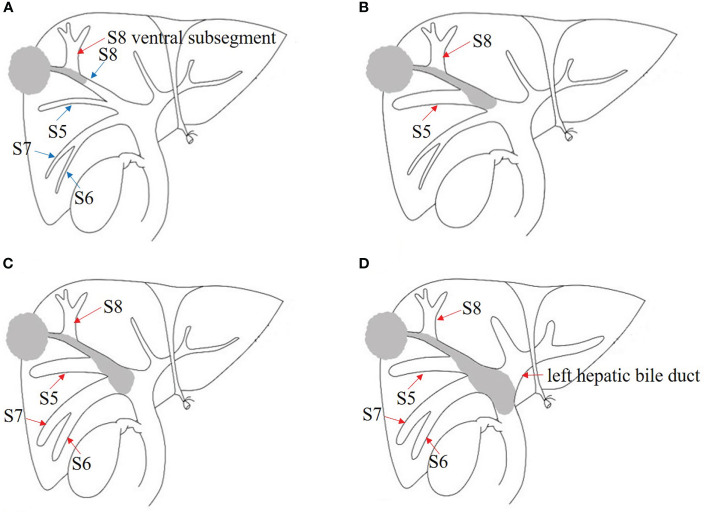
Japanese classification of BDTT. **(A)** B1: The tumor locates in S8 and the tumor thrombus invades the confluence of dorsal and ventral bile ducts; the bile duct dilation of ventral subsegment is observed. **(B)** B2: As the tumor thrombus further extends to the confluence of S5 and S8, bile duct dilation of S5 can be seen. **(C)** B3: The tumor thrombus extends to the right hepatic duct, and the bile duct dilation of the right posterior lobe can also be seen. **(D)** B4: The tumor thrombus extends to the common bile duct, and the bile duct dilation of left hepatic lobe can be seen. The bile duct dilation was marked with the red arrow.

Because few patients with BDTT have undergone surgical treatment at a single institution, this retrospective study was conducted at three high-volume institutions [12 in Fujian Provincial Hospital (Fuzhou, China), 8 in West China Hospital of Sichuan University (Chengdu, China), and 10 in the First Affiliated Hospital of Fujian Medical University (Fuzhou, China)]. From April 2010 to December 2019, 3,371 HCC patients underwent surgical treatment in the three institutions, and 112 (3.3%) patients were found to have BDTT. The diagnosis of HCC with BDTT was confirmed by the post-operative pathologic examination with two experienced pathologists. According to the classification proposed by the liver cancer study group of Japan, BDTT was classified as B1–B4 (**Figure 1**). The clinical data, imaging data, and pathological reports of 30 patients with B1–B3 BDTT and 631 patients without BDTT were recorded. A total of 631 patients without BDTT were randomly collected from each of the enrolled centers (230 in Fujian Provincial Hospital, 217 in West China Hospital of Sichuan University, and 184 in the First Affiliated Hospital of Fujian Medical University). From January 2020 to December 2020, the scans (CT or MRI) of 453 HCC patients (169 in Fujian Provincial Hospital, 143 in West China Hospital of Sichuan University, and 141 in the First Affiliated Hospital of Fujian Medical University) who underwent surgical treatment in the three institutions were collected for a reading test by blinded radiologists when using intrahepatic bile duct dilation in HCC patients as a potential biomarker for BDTT diagnosis. A total of six radiologists who did not know the clinicopathological information of the 453 HCC patients including original reports and pathological information were involved to report the scans. The six radiologists worked in pairs and all scans of the 453 HCC patients were reported by two radiologists who were in consensus. The results were compared with the original imaging diagnostic reports, which referred to the reports generated for the images when the scans were done. The present study was approved by the institutional review board of each institution.

### Image Acquisition

A 64-slice multidetector CT scanner (Toshiba, Aquilion, Japan) was used. The imaging study was performed from the diaphragm to the iliac crest. The scanning parameters were as follows: section thickness, 3 mm; tube voltage, 120 kV; tube current, 250 mA; and intersection gap, 5.0 mm. Iopromide (Ultravist 370, Bayer Schering Pharma, Berlin, Germany) was used as a contrast agent at a dose of 1.5 ml/kg, injection flow rate: 3–4.0 ml/s. After injection of contrast agent, HAP and PVP scans were performed at 34–37 s and 60–70 s, respectively.

MRI examinations were performed with a 1.5- or 3.0-T MRI system (Trio, Siemens Healthineers, Erlangen, Germany), using a torso coil. Transverse and coronal T1W scans were performed using the following sequences and parameters: breath-hold T1W fast low-angle shot sequence: TR, 170 ms; TE, 2.30/3.67 ms; flip angle, 150°; matrix size, 256 × 205; transverse T2W scan was performed using fat-suppressed turbo-spin-echo sequence: TR, 2,200 ms; TE, 103 ms; flip angle, 150°; matrix size, 320 × 106. The slice thickness was 5.0 mm with a 1.0-mm gap. All patients received power injector of 0.1 mmol/kg of body weight of gadopentetate dimeglumine (Magnevist, Schering, Berlin, Germany) *via* the antecubital vein at a rate of 2 ml/s. Serial dynamic contrast-enhanced scans were obtained on HAP (25–40 s), PVP (45–90 s), and equilibrium phase (2–5 min) after injection.

### Imaging Analysis

The imaging findings of HCC with BDTT were retrospectively analyzed as follows: background liver, tumor size, number of tumors, tumor capsule, the location of HCC lesions and BDTT, pre-contrast density and contrast enhancement characteristics of HCC lesions and BDTT, vascular tumor thrombus, intrahepatic metastasis or satellite nodule, and lymph node enlargement. Special attention was given to the presence or absence of intrahepatic biliary dilation. In comparison with background liver, the density of HCC and BDTT was divided as hypoattenuation, isoattenuation, or hyperattenuation in the pre-contrast, HAP, and PVP. All images were retrospectively and blindly reviewed by two senior abdominal radiologists in consensus.

### Pathology Analysis

HCC with BDTT was diagnosed based on histopathologic findings and immunohistochemical results. Macroscopically, the location, size, and capsule of HCC, presence of satellite nodules, necrosis or hemorrhage, vascular invasion, and the location and appearance of BDTT were observed. The histological differentiation of HCC with BDTT, microvascular invasion, lymph node metastasis, and liver cirrhosis were microscopically observed. The diagnoses and analyses were made by two experienced pathologists who were in consensus.

### Data Analysis

Statistical analyses were performed using SPSS software (version 17.0; SPSS, Inc., Chicago, IL, USA). Categorical variables were expressed as percentages and were compared using the chi-square test or Fisher’s exact test. Continuous variables were compared using *t*-test. *p* < 0.05 was considered statistically significant.

## Results

### Clinicopathological Characteristics

According to the Japanese classification, 5, 12, 13, and 82 patients in the present study were classified as B1, B2, B3, and B4, respectively (**Figure 1**). The incidence of HCC with BDTT was 3.3% (112/3,371), and B1–B3 BDTT accounted for 26.8% (30/112). The clinicopathological characteristics of HCC patients with B1–B3 BDTT and without BDTT are listed in **Table 1**. The two groups differed significantly in age, tumor number, portal vein invasion, lymph node metastasis, and tumor–node–metastasis (TNM) stage. No patients had obstructive jaundice before the operation.

**Table 1:** The clinicopathological feature of HCC patients with type B1–B3 BDTT and without BDTT.

Clinical information	B1–B3 BDTT (N = 30)	Without BDTT (N = 631)	*p*
Age (years)	48.5 ± 13.04	57.4 ± 12.32	<0.001
Gender			0.253
Male	23	533	
Female	7	98	
HBsAg			0.168
Positive	29	559	
Negative	1	72	
Background liver			0.555
No cirrhosis	3	87	
Cirrhosis	27	544	
Child-Pugh grade			0.634
A	28	601	
B	2	30	
Total bilirubin (µmol/L)	15.9 ± 4.15	15.3 ± 15.86	0.826
Albumin (g/L)	40.7 ± 5.64	42.34 ± 4.82	0.073
ALT(U/L)	51.8 ± 36.51	43.4 ± 38.53	0.239
AFP			0.096
Positive	27	486	
Negative	3	145	
Tumor number			<0.001
Single	11	512	
Multiple	19	119	
Tumor size (cm)	7.4 ± 3.05	6.9 ± 4.51	0.551
Capsule formation			0.225
Absent	25	569	
Present	5	62	
Portal vein invasion			<0.001
Yes	16	51	
No	14	580	
Lymph node metastasis			0.012
Positive	3	15	
Negative	27	616	
Tumor differentiation			0.479
Well+Moderate	12	294	
Poor	18	337	
TNM stage			<0.001
I/II	8	500	
III/IV	22	131	

HCC, hepatocellular carcinoma; BDTT, bile duct tumor thrombus; HBsAg, hepatitis B surface antigen; AFP, alpha fetoprotein; ALT, alanine aminotransferase; TNM, tumor–node–metastasis.

### CT and MRI Findings

**Figure 2:**
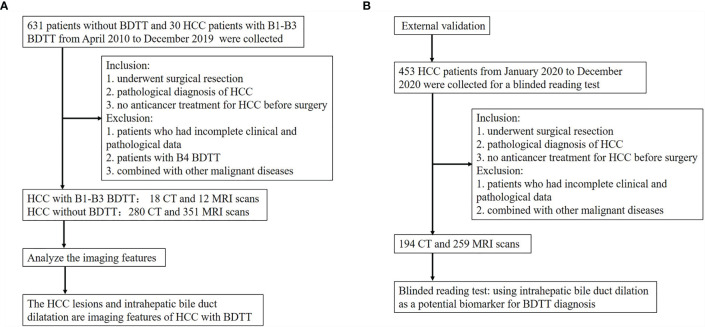
Flow diagram of the study. **(A)** The flow chart of analyzing the imaging features of HCC with B1–B3 BDTT. **(B)** The flow chart of the external validation.

In HCC with B1–B3 BDTT, 18 patients underwent CT and 12 patients underwent MRI scans. In HCC without BDTT, 280 patients underwent CT and 351 patients underwent MRI scans (**Figure 2A**). HCC lesions were detected in all patients. In HCC with B1–B3 BDTT, intrahepatic bile duct dilation was observed in 28 (93.3%) patients, while intrahepatic bile duct dilation was observed in 9 (1.43%) patients in HCC without BDTT. In the reports of the 30 HCC patients with B1–B3 BDTT, generated for the image when the scan was done, BDTT was observed in all B3 patients and 3 of 12 B2 patients, but it was not observed in B1 patients on CT or MRI.

**Figure 3:**
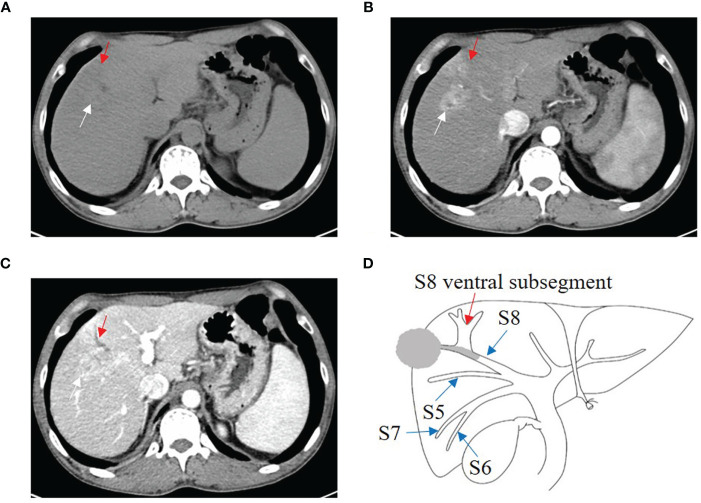
HCC with B1 BDTT. **(A)** The HCC lesion (white arrow) and bile duct dilation in S8 (red arrow) show hypoattenuation in plain CT image. **(B)** The HCC lesion has early enhancement (white arrow) in arterial phase, but enhancement of the bile duct dilation (red arrow) was not observed. **(C)** The HCC lesion (white arrow) shows hypoattenuation at portal venous phase, accompanied by dorsal bile duct dilation (red arrow) in S8. **(D)** HCC lesion, BDTT, and bile duct dilation (red arrow).

One B1 (**Figure 3**), 9 B2, and 8 B3 patients with BDTT underwent CT scans. The HCC lesions and BDTT showed relative hypoattenuation on plain CT scan, hyperattenuation at HAP, and hypoattenuation at PVP in all patients. Intrahepatic bile duct dilation showed no enhancement at the HAP and no progressively delayed enhancement at PVP, but it was more apparent in the PVP.

**Figure 4:**
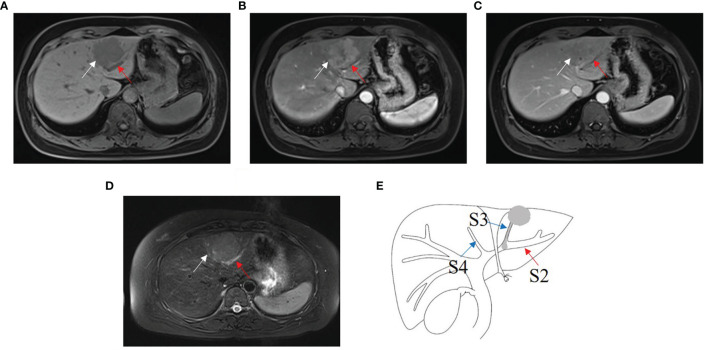
HCC with B2 BDTT. **(A)** HCC lesion (white arrow) in S3 and bile duct dilation (red arrow) in S2 show slightly hypoattenuation on T1W. **(B)** HCC lesion shows early enhancement (white arrow head) at hepatic arterial phase. **(C)** Rapid washout of contrast material (white arrow) at portal venous phase, accompanied by the bile duct dilation (red arrow) in S2. **(D)** HCC lesion (white arrow head) in S3 and bile duct dilation (red arrow) in S2 show hyperintensity on fat-suppressed T2W. **(E)** HCC lesion, BDTT, and bile duct dilation (red arrow).

**Figure 5:**
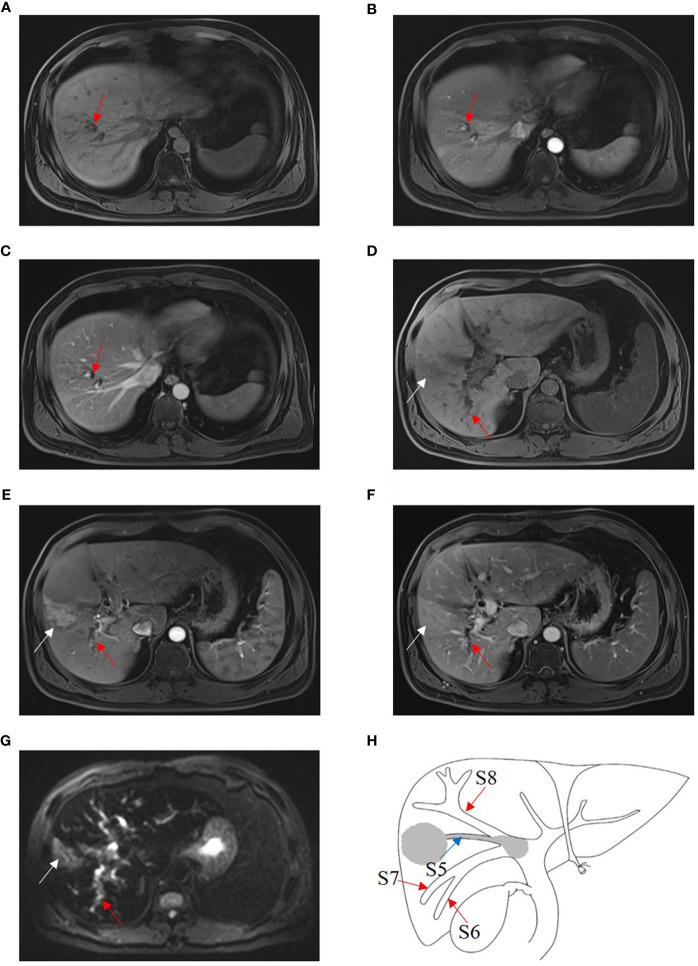
HCC with B3 BDTT. Bile duct dilation (red arrow) in S8 show slightly hypoattenuation on T1W **(A)**, arterial phase **(B)**, and portal venous phase **(C)**. HCC lesion locates (white arrow) in S5 and shows hypoattenuation in T1 phase **(D)**, enhancement (white arrow) at arterial phase **(E)**, and hypoattenuation (white arrow) at portal venous phase, accompanied by bile duct dilation (red arrow) in right posterior hepatic lobe **(F)**. **(G)** HCC lesion (white arrow) in S5 and bile duct dilation (red arrow) in the right posterior hepatic lobe show hyperintensity, and the bile duct tumor thrombus in the right hepatic duct shows hypointensity on fat-suppressed T2W. **(H)** HCC lesion, BDTT, and bile duct dilation (red arrow).

Four B1, three B2 (**Figure 4**), and five B3 BDTT patients (**Figure 5**) underwent MRI scans. The HCC lesions and BDTT showed relatively hyperattenuation on T2WI and relatively hypoattenuation on T1WI. Early enhancement of HCC lesions at HAP with hyperattenuation was observed, but thickened and grossly enhanced bile duct wall was not observed in all patients. At PVP, HCC lesions showed hypoattenuation in nine patients and isoattenuation in three patients, and intrahepatic bile duct dilation showed hypoattenuation in all patients.

**Figure 6:**
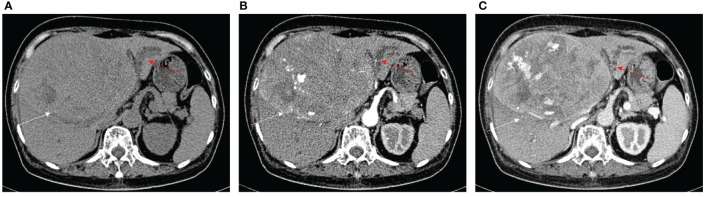
HCC without BDTT. **(A)** The HCC lesion (white arrow) and bile duct dilation in S2, S3, and S4 (red arrow) show hypoattenuation in plain CT image. **(B)** The HCC lesion has early enhancement (white arrow) in arterial phase, and bile duct dilation in S2, S3, and S4 (red arrow). **(C)** The HCC lesion (white arrow) shows hypoattenuation at portal venous phase, accompanied by closed bile duct dilation (red arrow) in S2, S3, and S4.

The imaging features of HCC with BDTT are summarized in **Table 2**. Intrahepatic bile duct dilation was observed in nine HCC patients without BDTT. Five patients underwent CT (**Figure 6**) and four patients underwent MRI scans. Of these patients, intrahepatic bile duct dilation was observed in S2, S3, and S4 (four patients); S4 (one patient); S6 and S7 (one patient); and S5 and S8 (three patients).

**Table 2:** Imaging findings of 30 HCC patients with type B1–B3 BDTT.

Variables	Values			
No.	Location of tumor	Location and type of BDTT		Dilation of bile duct
1	S5, S8	RAHBD	B2	S5, S8
2	S3	S3	B1	S3
3	S5	RAHBD	B2	S8
4	S3	LLHBD	B2	S2
5	S5, S8	RAHBD	B2	S5, S8
6	S2, S3	LHD	B3	S4
7	S5	RHD	B3	S6, S7, S8
8	S3	LLHBD	B2	S2
9	S2, S3	LLHBD	B2	S2, S3
10	S6, S7	RHD	B3	S5, S6, S8
11	S2	LLHBD	B2	S3
12	S2, S3	LHD	B3	S3, S4
13	S2	LLHBD	B2	S3
14	S2	LHD	B3	S3,S4
15	S5, S8	RHD	B3	S5, S6, S7, S8
16	S2	LLHBD	B1	S2
17	S5	RAHBD	B2	S8
18	S6	S6	B1	No
19	S5	S5	B1	No
20	S3	LHD	B3	S2, S4
21	S6	RPHBD	B2	S6, S7
22	S2	S2	B2	S2, S3
23	S8	RAHBD	B2	S5, S8
24	S8	RHD	B3	S5, S6, S7
25	S8	VBD of S8	B1	DBD of S8
26	S5, S8	RHD	B3	S6, S7, S8
27	S6	RHD	B3	S5, S7, S8
28	S3	LHD	B3	S2, S4
29	S7	RHD	B3	S5, S6, S8
30	S5, S8	RHD	B3	S6, S7

HCC, hepatocellular carcinoma; BDTT, bile duct tumor thrombus; DBD, dorsal bile duct; LHD, left hepatic duct; LLHBD, left lateral hepatic bile duct; RAHBD, right anterior hepatic bile duct; RHD, right hepatic duct; RPHBD, right posterior hepatic bile duct; S, segment; VBD, ventral bile duct.

### Intrahepatic Bile Duct Dilation in HCC Patients for BDTT Diagnosis

Intrahepatic bile duct dilation in CT and MRI scans was used for separating HCC patients with B1–B3 BDTT from HCC patients without BDTT. As it is shown in **Table 3**, intrahepatic bile duct dilation in HCC patients gives a better result for BDTT diagnosis. The sensitivity and specificity were 93.33% and 98.57%, respectively (**Table 4**). The positive predictive value and negative predictive value were 90.32% and 99.68%, respectively (**Table 4**).

**Table 3:** Intrahepatic bile duct dilation in HCC patients for BDTT diagnosis.

		HCC with B1–B3 BDTT	HCC without BDTT	Total
Intrahepatic bile duct dilation	Positive	28	9	37
	Negative	2	622	624
Total		30	631	

HCC, hepatocellular carcinoma; BDTT, bile duct tumor thrombus.

**Table 4:** Accuracy of using intrahepatic bile duct dilation in HCC patients for BDTT diagnosis.

Variable	Value
Sensitivity	93.33%
Specificity	98.57%
Positive predictive value	90.32%
Negative predictive value	99.68%

HCC, hepatocellular carcinoma; BDTT, bile duct tumor thrombus.

### Results of Blinding Test

A reading test by blinded radiologists was performed (**Table 5**). When using intrahepatic bile duct dilation in HCC patients as a potential biomarker for BDTT diagnosis by blinded radiologists in all 453 HCC patients (**Figure 2B**), 14 patients were classified as HCC with BDTT (7 as B1–B3 and 7 as B4, respectively). However, the original diagnostic reports showed that only three patients and seven patients were classified as B1–B3 and B4, respectively. Four patients with B1–B3 were misdiagnosed. More importantly, the diagnosis of all 453 HCC with or without BDTT by the postoperative pathologic examination was the same as the blinded reading test. The accuracy rate of diagnosis is 100%.

**Table 5:** A reading test was performed by blinded radiologists for BDTT diagnosis.

	Diagnosis by blinded radiologists	Original diagnostic reports	The pathological diagnosis
Without BDTT	439	443	439
With B1–B3	7	3	7
With B4	7	7	7
Total	453	453	453

BDTT, bile duct tumor thrombus.

## Discussion

Some studies found that large lesions, capsule infiltration, poor differentiation, portal vein invasion, and intrahepatic metastasis were more frequently observed in HCC patients with BDTT (5, 18, 19). These differences suggested that patients with BDTT had a more infiltrative nature, which accounted for poorer prognosis than those without BDTT, even after curative resection (7–9, 18–21). Since the bile duct and portal vein are encapsulated in the Glissonian sheath, tumors can easily involve both. About 46.7% of patients with BDTT had gross PVTT, and 73.3% were in advanced stages in the present study. In addition, patients with HCC and BDTT who underwent hepatectomy had a higher proportion of poorly differentiated tumors, lymphovascular invasion, and macrovascular invasion through systematic review and meta-analysis (7–9, 18–21). In our data, we also found that HCC patients with B1–B3 BDTT had more advanced tumor stages and adverse clinicopathological features. The two groups differed significantly in age, tumor number, portal vein invasion, lymph node metastasis, and TNM stage. For B4 BDTT, Kim et al. had shown that HCC patients with B4 BDTT had a higher incidence of jaundice, major vascular invasion, and a later AJCC stage (10). We also found that for HCC patients with B4 BDTT, there were significant differences in age, tumor number, portal vein invasion, lymph node metastasis, and TNM stage like B1 to B3 BDTT (data no showed).

Surgical treatment for HCC is considered the most effective approach, including those with BDTT. In addition, Kasai et al. had shown that extended hepatectomy for HCC patients with BDTT provided a better prognosis (6). Major liver resection and anatomical liver resection may be more suitable for patients with HCC and BDTT because it can remove HCC lesions, BDTT, and PVTT at the same time, improving R0 resection (10, 22–25). Luo et al. also showed that radical hepatic resection and removal of BDTT, combined with TACE, are the most effective approach for HCC patients with BDTT (26). These results showed that the choice of the most appropriate treatment is very important for the prognosis of HCC with BDTT. However, misdiagnosis of BDTT may lead to inappropriate therapeutic strategies before surgery, resulting in a poor prognosis. Moreover, Lu et al. had indicated that modification of the BCLC system to include BDTT might further enhance its prognostic ability (27). BDTT was associated with significantly worse long-term surgical outcomes in HCC patients (5, 20). Therefore, the early diagnosis of B1–B3 BDTT might help to choose suitable therapeutic strategies for patients and predict prognosis before surgery.

Despite recent remarkable improvements in imaging techniques, the diagnosis of HCC with BDTT remains a challenge. Patients with B1–B3 BDTT usually have no specific clinical manifestations and do not develop obstructive jaundice. In addition, both clinicians and radiologists are mostly satisfied with the diagnosis of HCC and lack sufficient awareness of BDTT. Of 34 patients with HCC and BDTT, all patients with B1–B3 BDTT and half of 24 patients with B4 BDTT were not diagnosed on preoperative CT or MRI scans (2). Ikenaga et al. reported that preoperative diagnosis of BDTT was obtained in 7 of 15 HCC patients with BDTT, but none of the 5 patients with B1, and 3 of 6 patients with B3 BDTT were not diagnosed preoperatively (18). Only 1 of 13 patients with B3 BDTT and none of the patients with B1–B2 BDTT were diagnosed before surgery in our study.

Therefore, distinctive imaging features of HCC with BDTT seem especially important to recognize. HCC lesions and soft tissue masses in the biliary ducts are two typical features, which is the key for diagnosing HCC with B4 BDTT (15, 17). In our data, although B1–B2 BDTT was not observed on CT or MRI scans, intrahepatic bile duct dilation was present in 93.3% of patients and indirectly reflected the presence of BDTT in the study. The tumor invades the bile duct of the subsegment, and bile duct dilation may not be detected on imaging. For example, if the tumor is located in S8 and the tumor thrombus extends to the dorsal bile duct, it does not invade the confluence of the dorsal and ventral bile ducts, and the bile duct dilation of the ventral segment may not be observed on CT or MRI scans. When the tumor thrombus invades the confluence of the dorsal and ventral bile ducts, bile duct dilation of the ventral subsegment is observed. As the tumor thrombus further extends to the confluence of S5 and S8, bile duct dilation of S5 can be seen. The tumor thrombus further extends to the right hepatic duct, and bile duct dilation of the right posterior lobe can also be seen. In HCC patients without BDTT, only 1.43% patients had intrahepatic bile duct dilation in CT or MRI scans. Of the patients, intrahepatic bile duct dilation was caused by the oppression of tumor. Therefore, intrahepatic bile duct dilation in HCC patients without BDTT was always closed to tumor. Our results confirmed that HCC lesions and the localized bile duct dilation may be imaging features of patients with B1–B3 BDTT.

Both CT and MRI have diagnostic value for HCC with BDTT, but MRI displays more detailed information for the diagnosis. Intrahepatic bile duct dilation can be seen in each phase of MRI, but it is more obvious in PVP on CT scans. When intrahepatic bile duct dilation in CT and MRI scans was used for separating HCC patients with B1–B3 BDTT from HCC patients without BDTT, the accuracy rate of diagnosis is 100% in the blinded reading test. Therefore, a deeper understanding imaging features of different BDTT is key to further improving the diagnosis preoperatively.

HCC with BDTT should be differentially diagnosed with intrahepatic cholangiocarcinoma (intraductal type). Both HCC with BDTT and intrahepatic cholangiocarcinoma have similar image features, such as intraductal neoplasm and upstream bile duct dilation (17, 28). Most BDTT show early enhancement at HAP and rapid washout of contrast agent with hypointense signal at PVP (14, 29). Intrahepatic cholangiocarcinoma usually manifests as a narrowed bile duct with irregular wall thickening and progressively delayed enhancement of the PVP (17). Hepatic parenchymal mass and the T1W hyperintense signal on the distal segment are valuable for distinguishing BDTT from intraductal growing cholangiocarcinoma (28). The presence of liver cirrhosis, serum CA19-9, and AFP levels are also supportive of the differential diagnosis. Another relatively rare disease, but also to be distinguished from BDTT, is the HCC compressing the intrahepatic bile duct. The latter can cause intrahepatic bile ducts to dilate, and the location of bile duct dilation is where the HCC compresses the bile duct. However, bile duct dilation in HCC patients with BDTT is caused by tumor thrombus, not the tumor itself. The tumor and the dilated bile duct have a certain distance, rather than close to the dilated bile duct.

Several limitations of our study need to be acknowledged. First, our study had a relatively small sample size due to the rare incidence of these tumors. Despite this, our population is the largest among the published studies. Second, because there was no jaundice, none of the patients with BDTT received MRCP before the operation. Thus, to some extent, our explanations for the imaging findings of B1–B3 BDTT might be considered speculative before operation.

## Conclusion

In summary, HCC lesions and intrahepatic bile duct dilation on CT or MRI scans were commonly seen in HCC patients with B1–B3 BDTT. These imaging features facilitate the early diagnosis of B1–B3 BDTT, which might help to choose suitable therapeutic strategies for patients before surgery.

## Data Availability Statement

The original contributions presented in the study are included in the article/supplementary material. Further inquiries can be directed to the corresponding authors.

## Ethics Statement

The studies involving human participants were reviewed and approved by Fujian Provincial Hospital (Fuzhou, China), West China Hospital of Sichuan University (Chengdu, China), and the First Affiliated Hospital of Fujian Medical University (Fuzhou, China). The patients/participants provided their written informed consent for participation in this study. Written informed consent was obtained from the individual(s) for the publication of any potentially identifiable images or data included in this article.

## Author Contributions

Jun-YW: Methodology, Software, Resources, Data curation, and Writing original draft. L-MH: Methodology, Software, Resources, and Writing original draft. Jia-YW: Data curation, Formal analysis, and Methodology. Y-GW: Data curation, Supervision, and Validation. Z-BZ: Investigation, Methodology, and Project administration. Y-NB: Conceptualization, Supervision, and Validation. M-LY: Conceptualization, Supervision, Validation, and Writing original draft. All authors contributed to the article and approved the submitted version.

## Funding

This research was supported by the Natural Science Foundation of Fujian Province, China (Grant No. 2020J011105).

## Conflict of Interest

The authors declare that the research was conducted in the absence of any commercial or financial relationships that could be construed as a potential conflict of interest.

## Publisher’s Note

All claims expressed in this article are solely those of the authors and do not necessarily represent those of their affiliated organizations, or those of the publisher, the editors and the reviewers. Any product that may be evaluated in this article, or claim that may be made by its manufacturer, is not guaranteed or endorsed by the publisher.
